# Characteristics of Successful Community Partnerships to Promote Physical Activity Among Young People, North Carolina, 2010–2012

**DOI:** 10.5888/pcd10.130110

**Published:** 2013-12-12

**Authors:** Joni D. Nelson, Justin B. Moore, Christine Blake, Sara F. Morris, Mary Bea Kolbe

**Affiliations:** Author Affiliations: Joni D. Nelson, Christine Blake, University of South Carolina, Columbia, South Carolina; Sara F. Morris, Mary Bea Kolbe, North Carolina Department of Health and Human Services, Raleigh, North Carolina.

## Abstract

**Introduction:**

Success of community-based projects has been thought to hinge on the strength of partnerships between those involved in design and implementation. However, characteristics of successful partnerships have not been fully described, particularly in the context of community-based physical activity promotion. We sought to identify characteristics of successful partnerships from the perspective of project coordinators involved in a mini-grant program to promote physical activity among young people.

**Methods:**

Semistructured qualitative interviews were conducted with county coordinators (n = 19) of 20 North Carolina’s “Eat Smart, Move More” Community Grants projects funded during 2010 through 2012. Emergent themes were coded; then, overarching themes in the coded data were identified and grouped with similar codes under thematic headings. On the basis of project coordinators’ responses, each partnership was classified as strong, moderate, or weak.

**Results:**

Three overarching themes characterized partnership relationships: continuity (history with partner and willingness to engage in a future partnership), community connectedness, and capacity (interest, enthusiasm, engagement, communication, and clarity of roles and responsibilities). Strong partnerships were those in which project coordinators indicated a positive working history with partners, experienced a high level of engagement from partners, had clearly defined roles and responsibilities of partners, and expressed a clear interest in working with their partners in the future.

**Conclusion:**

In community partnerships aimed at increasing physical activity among young people, the perspectives of project coordinators are vital to identifying the characteristics of strong, moderate, and weak partnerships. These perspectives will be useful for future community program development and will influence potential health outcomes.

## Introduction

Many community interventions to promote physical activity among young people involve partnerships between public health organizations focused on health promotion (1–4). Funding opportunities administered through the Centers for Disease Control and Prevention require coordinated participation with local public health stakeholders. Prior studies on partnership quality suggest that member involvement and satisfaction are vital to the success of partnerships (5). Evidence shows a relationship between collaborative community partnerships and outcomes (6) but also suggests it can be difficult to evaluate the cohesiveness of research partnerships and to demonstrate a causal relationship between community collaboration and a project’s success (7). Positive perspectives from program coordinators and future engagement with partners were key factors to successful partnerships within that study. Therefore, we aimed to examine varying characteristics of partnership strength to further explore the success of partnerships. Partnerships perceived as “strong,” in comparison with those perceived to be “weak,” may be more capable of increasing physical activity among young people, decreasing the likelihood of future adult chronic disease (eg, cardiovascular disease, mental health problems, diabetes). Funding for interventions to increase physical activity among young people is usually short-term, therefore necessitating strong partnerships for their success. Features of strong versus weak partnerships need to be identified in order to properly allocate funds to groups likely to maintain successful partnerships during future programs and to produce positive outcomes.

We describe characteristics of strong partnerships from the perspective of community project coordinators who are promoting physical activity among young people as part of after-school and school-based programs in 20 North Carolina counties.

## Methods

In 2010, as part of an evaluation of the effectiveness of the “Eat Smart, Move More” (ESMM) Community Grants Program, 20 North Carolina county health departments were selected, through a competitive application process, to receive grants supporting projects aimed at increasing physical activity among young people aged 9 to 14 years. Projects were based in both schools and after-school programs.

Participants in this study were county coordinators (n = 19) of the 20 ESMM Community Grants projects selected for funding during 2010 through 2012. One county project coordinator was responsible for coordinating 2 of the 20 projects because the 2 counties were part of the same health district. All county project coordinators were women.

Semistructured qualitative interviews were conducted (2011–2012) with project coordinators from each of the 20 counties. Interviews were conducted via telephone by a trained interviewer. The semistructured interview guide included a core set of questions for all participants and a set of questions tailored to each participant’s responses to a project coordinator self-assessment and another electronic survey (both administered at an earlier time) (Table 1). Questions were related to project coordinators’ prior grant coordinating experiences, partnership work relations, barriers to partnership engagement, perceived growth as a result of working on this grant, and perception of project success. Interviews lasted 30 to 45 minutes and were audio recorded. Audio recordings were transcribed verbatim and analyzed using thematic qualitative data analysis and the constant comparative method (8–10).

**Table 1:** Sample of Interview Questions Tailored to Project Coordinators, North Carolina, 2010–2012

Project Coordinator Interview Questions	Question Aim
How long have you been in your primary position? How often do you work with *{name data collection/project sites, personalize by county}*?	Project coordinators’ prior grant coordinating experiences
Which of these partners *{name each participating partner}* would you work with again on another project?	Partnership work relations
What do you think contributed to/facilitated *{specific partner’s/stakeholder’s}* engagement being more than what you expected? What do you think were the barriers that contributed to *{specific partner’s}* engagement being less than what you expected?	Barriers to partnership engagement
Was there any particular aspect of coordinating this specific grant project that you think contributed to the change in your assessment of your skills?	Perceived growth as a result of working on this grant
On a scale of 1 to 5, where 1 is not successful at all and 5 is very successful, how would you rate this project’s success at impacting *{name target population according to response above}*, as planned? Is there anything you would do differently to improve the impact of the project on the target population?	Perception of project success

Data analysis followed procedures based on the constant comparative method in continuous data analysis (11), and steps were taken to ensure the trustworthiness of findings. Analytic steps were as follows: 1) we developed a preliminary code book that best represented project coordinators’ reported experiences and was guided by the aims of the research; 2) two experienced qualitative researchers independently coded 3 interviews using the preliminary code book and developed additional codes based on emergent themes using qualitative data analysis software NVivo 9 (QSR International Pty Ltd, Doncaster, Victoria, Australia); 3) coding from the 3 interviews was compared; 4) page-by-page comparisons were conducted, and differences in application or new code development were discussed by the research team until consensus was reached; 5) modifications to the final code book were made; 6) one researcher used the final code book to code all 20 interviews; 7) selective coding was conducted to group similar subthemes (eg, partner interest, enthusiasm) in overarching representative themes (eg, capacity); and 8) matrices were developed to explore responses across sites and compare relationships, repetition of themes within an interview and across interviews, patterns of responses across participants, and differences in responses.

Characteristics of strong, moderate, and weak partnerships emerged from the perspectives of project coordinators, and classification was determined by examining matrices that allowed for grouping of sites by types of behaviors, activities, strategies, work relations, and experiences. For instance, when project coordinators expressed solely positive experiences, partnerships were characterized as strong. In contrast, if only challenges and barriers were described by project coordinators, these partnerships were characterized as weak. Partnerships were characterized as moderate when project coordinators described barriers to success and both positive and challenging experiences. The research team met biweekly to discuss the findings for interpretation in relation to the study aims and existing theoretical frameworks.

## Results

The experiences described by project coordinators involved in project partnerships to promote physical activity among young people were represented by 3 overarching themes, including major subthemes: continuity (subthemes: history with partner and willingness to engage in future partnership), community connectedness, and capacity (subthemes: interest, enthusiasm, engagement; communication; and clarity of roles and responsibilities). Each county was categorized as having a strong, moderate, or weak partnership based on patterns across all themes.

### Continuity

**Project coordinator’s history with partner**

History and experience with partners influenced current partnership quality. Project coordinators described previous collaborations and other types of relationships they had with their partners that caused them to value their current partnership. One project coordinator stated,

Over the past five years I guess I’ve worked with them on about two larger projects, one being this one . . . it’s a continued partnership that we have . . . I can’t really quantify it but . . . we work with them enough to value their partnership. (Interview 1)

Another project coordinator explained that having a history with the partner enhanced trust and shortened time required for action because relationships were already established. She said,

[We had] a good relationship with the community, a good relationship with the schools. So it was very easy to jump into this particular grant because I already knew everyone who was involved, and we already had that trust relationship built. (Interview 2)

In contrast, another project coordinator mentioned how previous interaction with a particular partner caused her to expect less engagement from that partner during project implementation.

We worked on other projects together, but it was a matter of, well I didn’t know how much time they have, they’re both directors, and sort of big shots in their organizations. I don’t want to get my hopes up too much. (Interview 3)

**Willingness to engage in a future partnership**

Many project coordinators discussed their willingness or unwillingness to engage in future partnerships with their current partners. Future partnership was described as the potential occurrence of the project coordinator continuing their working relationship with their partners for additional projects. Project coordinators expressed willingness to engage in future work with these partners as a reflection of their overall assessment of partnership quality. For example, 1 project coordinator stated,

He’s been marvelous to work with. I think he’s going to be a very strong community partner into the future. Our relationship’s really good. (Interview 4)

Difficulties with a partnership also shaped a project coordinator’s perception about future collaboration and conditions under which they would be willing to enter into a new partnership. One project coordinator stated,

I think they’re a great partner and we’re going to continue working with them, but I just need to be a better navigator . . . from now on. (Interview 5)

### Community Connectedness

The project coordinators provided insight into how well their partners used their local and community resources for project purposes. Project coordinators described how their partners’ reputation within the community was an asset to the partnership. One project coordinator stated that her partner had a “good reputation with community partners, previous successes with partners” (Interview 2) and that this history of success and connection to the community permitted access to certain assets (eg, networking with community leaders) that the project coordinator would not have had otherwise. Another project coordinator stated,

It was nice to have that connection; as far as she went with and introduced us to the people we needed to know. (Interview 6)

### Capacity

**Partner interest, enthusiasm, and engagement**

All project coordinators discussed their partners’ level of interest and enthusiasm, which influenced how involved the partners were during implementation and their engagement throughout the partnership. Some project coordinators talked about how their partners were “really passionate about [the project]” (Interview 6) and that their partners were “at every conversation [at which they were] needed” (Interview 7).

Some project coordinators described how energetic their partners were throughout the duration of the study and how this energy reflected sustained motivation. Conversely, some project coordinators said that their partners seemed to grow tired of the study when they had to wait for a long time between initial involvement and implementation. One project coordinator stated,

I mean she was involved but, I think when we first wrote the grant she was really excited about having that year in between us, getting the grant and then implementing the grant committees . . . I guess her steam kinda . . . lessened . . . I mean she was still really involved, she was just, was so excited I kinda expected more. (Interview 8)

Some project coordinators described unexpected disruptions that negatively affected certain partners’ ability to maintain engagement with the project. These included frequent partnership replacements, commitment to other projects, and job changes. One project coordinator described the experience of trying to adjust to working with a new partner in the middle of the project, saying,

In order to bring [them] onto the team or how, who’s going to . . . be able to fit that into their job responsibilities and stuff like that. So I think . . . that’s really been the only thing that’s been a little bit difficult. (Interview 9)

Other external barriers influenced partnerships, such as unexpected trauma in partners’ personal lives (ie, family death or illness). For example, 1 project coordinator described the difficulty of working with a partner who was overburdened with personal tragedy, stating, “[the partner] really couldn’t devote the time that he had thought that he would be able to put into the project . . . [he] lost both of his parents this year” (Interview 8).

**Partners’ communication**

The quality of communication provided insight into the partners’ level of engagement with the project. The project coordinators described good communications about meeting times, trainings, and various aspects of the study as indications of the overall quality of the partnership. One project coordinator stated,

We've been able to develop partnerships that foster regular and easy communication. Without this easy, open communication we would not be able to stay on the same page and motivate each other toward project success. (Interview 10)

In contrast, another project coordinator revealed poor communication by stating,

It’s probably a lot that . . . she is really involved in another program. I think what happened was a lot of time I would phone or I would e-mail, and I would not get a response back . . . [and] I don’t know. But I think I got real frustrated, because I was not getting responses back that I thought I should. (Interview 11)

**Clarity of roles and responsibilities**

Project coordinators discussed the importance of having specific roles and responsibilities for the grant project, for both themselves as the project coordinator and their partners. Roles and responsibilities were described as the specific duties and level of accountability designated to either project coordinators or partners for the duration of the study. One project coordinator stated,

We are able to talk through barriers and assign responsibilities to help mitigate those concerns without overly taxing any member of our group. (Interview 10)

Project coordinators described their roles and the roles they expected their partners to take in relation to project implementation. Additionally, 1 project coordinator described how some of their partners did “not fully understand their role” (Interview 5). These misunderstandings ultimately led to some problems with project implementation.

### Partnership strength

On the basis of project coordinators’ self-reported assessment of the overall strength of their respective partnerships and experiences with their current partnerships, we classified 8 of the 20 partnerships as strong, 9 as moderate, and 3 as weak (Table 2).

**Table 2:** Characteristics of Strong, Moderate, and Weak Community Partnerships, as Assessed by Project Coordinators, North Carolina, 2010–2012

Characteristic	Strong	Moderate	Weak
**History with partner**	● Project coordinators had worked with their partner before project implementation ● A working relationship was, in some capacity, previously established between partners	Project coordinators had prior knowledge about their partners from previous study project successes, but did not express any sentiments about prior experience with their partners	● No prior experience working with partners ● New relationships were built during project development and implementation
**Partner reputation and community connectedness**	Project coordinators had prior positive impressions about their partners’ reputation and community connectedness	Project coordinators had prior positive impressions about their partners’ reputation but limited information about community connectedness	Project coordinators’ descriptive commentary did not clearly associate their experience with partner reputation and community connectedness^a^
**Partner interest, enthusiasm, and engagement**	● Partners took a serious interest in the project ● Partners actively involved ● Partners expressed passion for project implementation ● Partners had a high level of engagement, worked well with their project coordinators throughout the duration of the study	● Partners took a serious interest in the project ● Partners actively involved, but considered a moderate level of engagement ● Project coordinators worked well with their partners, but some difficulties related to partner replacements and other external barriers	● Project coordinators did not always experience high levels of partnership engagement ● Partners not always available ● Partners did not fully understand the details and actions needed for project implementation ● Frequent difficulties with partners ● Partnership replacements ● Personal complications with partners
**Clarity of roles and responsibilities**	Roles and responsibilities for partners and project coordinators were adequately defined and fulfilled throughout the duration of the project	● Duties and roles not always fulfilled by partners ● Roles and responsibilities held between partners not clearly defined	● Partnership disorganization ● Roles not defined or understood throughout duration of the project ● Duties and responsibilities of partners not fulfilled
**Willingness to engage in a future partnership**	Project coordinators expressed no doubt about working with their partners in the future	Project coordinators expressed some hesitance in regard to working with their partners in the future	Project coordinators expressed doubt and hesitance about working with their partners in the future

a Weak partnership was not reflected in the data regarding partner reputation and community connectedness.

Strong partnerships were those in which project coordinators indicated a positive working history with partners, experienced high level of engagement from partners, had clearly defined roles and responsibilities for partners, and expressed an interest in working with their partners in the future. Often project coordinators in strong partnerships were eager to express their sentiments regarding their partners and expressed immense interest in working with their partners in the future, as in the case of the project coordinator who said, “I would [work] with all of them [again], if given the opportunity” (Interview 12).

In moderate partnerships, project coordinators had not worked directly with their partners before, indicated that roles and responsibilities were not clearly defined, and expressed subtle hesitance with regard to working with their partners in the future. Project coordinators with partnerships characterized as moderate had minimal issues with their partners but often suggested how they could improve their partnerships.

I think it was just a matter of over time we were able to develop better relationships and understand communication channels that were preferred for each of the partners, and how they would like to be given information, or share information. (Interview 3)

Weak partnerships were those in which project coordinators had no prior working experience with their partners, had difficulty clarifying duties and roles, and had less than desirable levels of engagement with partners. Among those project coordinators who described weak partnerships, there was hesitancy to directly criticize or label issues that contributed to the weak partnership. Problems with the partnership were presented with subtlety with statements such as,

I’m not saying I would not work with them again. I would. It’s just difficult at times. (Interview 11)

## Discussion

Most project coordinators saw partnership continuity as an important feature of a successful partnership. This finding is consistent with findings of a previous study that examined the relationship between local health directors’ partner characteristics and partnership effectiveness (12) and found that time engaged as partners was a significant predictor of effectiveness. However, this is the first study to report that willingness to engage in future partnership is an important characteristic of continuity that should be considered when evaluating partnerships.

The continuity of the partnerships was based in part on the ability of partners to remain actively engaged. Recurring partner turnover, poor quality of communication with partners, and difficulties with clarification of partners’ roles are characteristics that decrease partnership engagement and lead to a weak partnership and a low level of partnership success. The importance of having partners who can consistently fulfill day-to-day project duties strongly suggests the need for built-in redundancy across roles and responsibilities (ie, partners should train others within their agency to adopt the responsibilities of the project). Although a limitation for certain community organizations, this recommendation may mitigate the debilitating effects of partnership loss or a change in partners.

Connectedness of the partner to community resources was seen as an important characteristic of strong partnerships. This finding suggests that project-specific partnerships might function like large-scale coalitions. Large-scale network analyses suggest that connectedness is a key feature of valuable coalition members engaged in physical activity promotion (13). This finding was especially true for the participants of this study, since the ESMM Community Grants request for applications specifically calls for environmental approaches to physical activity promotion among young people, and environmental approaches often involve engaging diverse stakeholders (eg, after-school or school administrators, teachers, parents).

This study has some limitations. Data presented come exclusively from the county grant project coordinators. Community partners of the project coordinators may have different perceptions of the partnership that should be considered. No existing criteria gauge partnership strength; thus, we are unable to compare our results against a gold standard. The project coordinators who participated within the study are intimately invested in the projects around which these partnerships are built. The project coordinators’ self-interest in revealing characteristics of a quality partnership may weigh deeply on responses provided within the interviews (ie, project coordinators’ self-interest may elicit positive responses that are not completely reflective of actual partnership experiences).

Although these limitations should be considered, our study has numerous strengths. Participants shared their experiences by using inductive qualitative methods, which provided insights that may not have been identified by using more structured approaches (eg, dichotomous survey). The interviewer was indirectly familiar with North Carolina’s county physical activity programs for young people, which allowed the interviewer to ask targeted questions. Data from the project coordinators were rich and varied, which allowed us to characterize partnerships as strong, moderate, or weak.

This study demonstrates that broad themes of continuity, connectedness, and capacity appear to be key elements of strong community partnerships for promoting physical activity among young people. These findings are important, because they describe features of collaborations that should be considered when making funding decisions. Although these are not the only features of partnerships that should be considered, these characteristics identified by project coordinators may be the most easily assessable and informative. Future studies should examine how these key elements relate to program outcomes and partnership sustainability for future program development.
